# Identification by MALDI-TOF MS of *Sporothrix brasiliensis* Isolated from a Subconjunctival Infiltrative Lesion in an Immunocompetent Patient

**DOI:** 10.3390/microorganisms8010022

**Published:** 2019-12-21

**Authors:** Aline M. F. Matos, Lucas M. Moreira, Bianca F. Barczewski, Lucas X. de Matos, Jordane B. V. de Oliveira, Maria Ines F. Pimentel, Rodrigo Almeida-Paes, Murilo G. Oliveira, Tatiana C. A. Pinto, Nelson Lima, Magnum de O. Matos, Louise G. de M. e Costa, Cledir Santos, Manoel Marques Evangelista Oliveira

**Affiliations:** 1 Department of Ophthalmology, University Hospital of the Federal University of Juiz de Fora, Juiz de Fora 36038-330, Brazil; alinemotafreitas@yahoo.com.br (A.M.F.M.); biancabarczewski@hotmail.com (B.F.B.); lucas_xavier_jf@hotmail.com (L.X.d.M.); vargasjordane@yahoo.com.br (J.B.V.d.O.); 2 Laboratório de Micologia, Instituto Nacional de Infectologia Evandro Chagas, Fundação Oswaldo Cruz, Rio de Janeiro 21040-900, Brazil; l.machadomoreira@gmail.com (L.M.M.); rodrigo.paes@ini.fiocruz.br (R.A.-P.); 3 Laboratório de Pesquisa Clínica e Vigilância em Leishmanioses, Instituto Nacional de Infectologia Evandro Chagas, Fundação Oswaldo Cruz, Rio de Janeiro 21040-900, Brazil; maria.pimentel@ini.fiocruz.br; 4 Department of Pharmacy, Federal University of Juiz de Fora, Juiz de Fora 36036-900, Brazil; murilogo@hotmail.com; 5 Instituto de Microbiologia Paulo de Goes, Universidade Federal do Rio de Janeiro, Rio de Janeiro 21941-901, Brazil; tati.micro@gmail.com; 6 CEB—Biological Engineering Centre, University of Minho, Campus de Gualtar, 4710-057 Braga, Portugal; nelson@ie.uminho.pt; 7 Imaging Department of Instituto Oncológico, Hospital Nove de Julho, Juiz de Fora 36010-510, Brazil; magnummatos@yahoo.com.br; 8 Department of Pathology, Faculty of Medicine of Federal University of Juiz de Fora, Juiz de Fora 36038-330, Brazil; louisepatologia@gmail.com; 9 Department of Chemical Science and Natural Resources, BIOREN-UFRO, Universidad de La Frontera, 4811-230 Temuco, Chile; 10 Laboratório de Pesquisa Clínica em Dermatozoonoses em Animais Domésticos, Instituto Nacional de Infectologia Evandro Chagas, Fundação Oswaldo Cruz, Rio de Janeiro 21040-900, Brazil; 11 Laboratório de Taxonomia, Bioquímica e Bioprospecção de Fungos, Instituto Oswaldo Cruz, Fundação Oswaldo Cruz, Rio de Janeiro 21040-900, Brazil

**Keywords:** sporotrichosis, *Sporothrix brasiliensis*, ocular sporotrichosis, fungal identification, MALDI-TOF MS

## Abstract

Sporotrichosis is a globally distributed subcutaneous fungal infection caused by dimorphic fungi belonging to the *Sporothrix* species complex that affects the skin of limbs predominantly, but not exclusively. A rare case of ocular sporotrichosis in an immunocompetent Brazilian patient from the countryside of Rio de Janeiro State is reported. A 68-year-old woman presented with a subconjunctival infiltrative lesion in the right eye with pre-auricular lymphadenopathy of onset 4 months ago that evolved to suppurative nodular lesions on the eyelids. Conjunctival secretion was evaluated by histopathological examination and inoculated on Sabouraud Dextrose Agar (SDA). Histopathology showed oval bodies within giant cells and other mononucleated histiocytes. Fungus grown on SDA was identified as *Sporothrix* sp. by morphological observations. The isolated strain was finally identified by Matrix-Assisted Laser Desorption/Ionization Time-of-Flight Mass Spectrometry (MALDI-TOF MS) associated with an in-house database enriched with reference *Sporothrix* complex spectra. The strain presented a MALDI spectrum with the ion peaks of the molecular mass profile of *S. brasiliensis*. The patient was adequately treated with amphotericin B subsequently replaced by itraconazole. Due to scars left by the suppurative process, the patient presented poor final visual acuity. The present work presents an overview of ocular sporotrichosis and discusses the diagnostic difficulty that can lead to visual sequelae in these cases.

## 1. Introduction

Sporotrichosis is a globally distributed subcutaneous fungal infection caused by the dimorphic fungi belonging to the *Sporothrix* species complex, which can affect animals and humans [1,2,3]. Particularly common in tropical and subtropical areas, this infection is caused by transcutaneous trauma involving animals, soil, plants, or organic matter contaminated with these fungal species through which the fungal conidia enter into the host [4,5]. Such infection may progress into chronic cutaneous and/or it may spread to internal organs [4,5].

The establishment of this mycosis in ocular adnexa is rare [1,6,7], and typically limited to the eyelids and eyebrows with accompanying regional lymphadenopathy following traumatic inoculation, or as fixed-cutaneous ulceration or conjunctivitis following exposure [6,8,9]. However, the number of reported ocular sporotrichosis cases has increased in recent years [6,7,8,10,11,12,13], especially in Rio de Janeiro State, Brazil, where sporotrichosis is endemic [14,15].

Fungal taxonomy has historically been based mainly on morphological traits. However, the use of fungal macro- and micromorphological traits is not enough to differentiate fungal interspecific similarities. Genotypic traits provide the greatest number of variable characters for fungal taxonomy. However, even with the high level of sensitivity and resolution associated with molecular methods, some problems still arise when molecular techniques are applied for the identification of fungal genetic close related species [16,17]. Nevertheless, DNA housekeeping genes sequencing remains the gold standard for fungal identification [18].

Each morphological and molecular technique presents limitations and advantages when applied to filamentous fungi identification. Both of them can be used as complementary methods for reliable fungal identification. More recently, proteomic profiles generated by Matrix-Assisted Laser Desorption/Ionization Time-of-Flight Mass Spectrometry (MALDI-TOF MS) have been used as an important tool for the identification of filamentous fungi isolated from different substrates [17,18,19,20,21,22].

For the use of MALDI-TOF MS in clinical settings as a reliable, fast, and cost-effective fungal identification system, an associated and accurate database is essential [22]. In this present work, a clinical case report of ocular sporotrichosis in a patient where the isolate was assessed by classical taxonomy and histopathology and, finally, identified by MALDI-TOF MS associated with an in-house database enriched with reference *Sporothrix* complex spectra, is presented. Moreover, a brief overview of previously reported cases is presented and discussed.

## 2. Materials and Methods

### 2.1. Case Report

A 68-year-old female patient, housewife, living in the city of Paty do Alferes in the South Region of Rio de Janeiro State, Brazil, with a history of systemic arterial hypertension and depression, presented with ocular inflammation 4 months ago, which evolved into a suppurative lesion in the right eye (RE), associated with ocular pain. She was examined by different specialists and prescribed with (1) oral antibiotics cephalosporins and beta-lactam (Cefalexin 500 mg each 6 h for 10 days, and amoxicillin/clavulanate 875/125 mg each 12 h for 7 days); (2) topical antibiotics (tobramycin eye drops 3 mg/mL 1 drop each 6 h for 7 days, and moxifloxacin eye drops 0.5% 1 drop each 6 h for 7 days) and, (3) antivirals (acyclovir 400 mg/day for 14 days). The response to the several treatments was null.

The patient was attended at the ophthalmology outpatient clinic of the University Hospital of the Federal University of Juiz de Fora (Juiz de Fora, Minas Gerais State, Brazil), where she underwent an ophthalmologic examination and reported a history of home contact with a cat that had skin lesions. The main diagnostic hypothesis of sporotrichosis was established, but other infiltrative and neoplastic causes needed to be ruled out. The patient was hospitalized to perform imaging exams (Nuclear Magnetic Resonance) of the ocular orbit and biopsy of the conjunctival lesions. The material from the biopsy was sent to pathological examination and conjunctival secretion was sent to microbiological culture.

### 2.2. Etiological Agent and Taxonomy

The specimen underwent routine examinations, which involved bacterioscopic and direct examination with potassium hydroxide, culture on Sabouraud Dextrose Agar 2%, and Mycosel Agar (both from Difco™, Sparks, NV, USA). Cultures were incubated at 25 °C and were observed over 4 weeks for fungal growth. Dimorphism was demonstrated by conversion to the yeast-like form on Brain Heart Infusion Agar (Difco™, Sparks, NV, USA) at 37 °C for 1 week. In addition, a slide culture was performed on Potato Dextrose Agar (Oxoid, Basingstoke, UK)), incubated at 25 °C for 7 days and mounted with Lactophenol Cotton Blue (Fluka, Buchs, Switzerland) for *Sporothrix* identification [1,23].

Identification at the species level was performed using MALDI-TOF MS as previously described by Oliveira and collaborators [16]. Briefly, 10^6^ yeast cells were transferred from the culture plate at the yeast phase (c.a. 1 μg) to a 500 μL tube containing 20 µL of 25% formic acid in water (*v*/*v*). The supernatant of each sample (1 μL) was transferred to a paraffin film surface and 2 μL of the matrix solution α-cyano-4-hydroxycinnamic acid (CHCA, Fluka, Buchs, Switzerland) was added and gently mixed. Each suspension (1 μL) was spotted onto the MALDI-TOF MS stainless plate (FlexiMass™, Shimadzu Biotech, Manchester, UK) in triplicate to test reproducibility. Previous to the spectra acquisition, the sample was air-dried at room temperature.

Spectra acquisition was performed on an Axima LNR equipment (Kratos Analytical, Shimadzu, Manchester, UK) equipped with a nitrogen laser (337 nm). Mass range from 2000 to 20,000 Da was recorded using the MALDI-TOF MS linear mode with a delay of 104 ns and an acceleration voltage of +20 kV. For the CHCA matrix, final spectra were generated by summing 2 laser shots accumulated per profile and 100 profiles produced per sample, leading to 200 laser shots per summed spectrum.

The resulting peak lists were exported into the SARAMIS^®^ software package (Spectral Archiving and Microbial Identification System; AnagnosTec, Postdam-Golm, Germany) for data analyses, and final microbial identification was achieved. Peak lists of individual samples were compared to the SARAMIS™ database, enriched with in-house spectra of reference strains of the *Sporothrix* species complex, generating a ranked list of matching spectra.

### 2.3. In Vitro Susceptibility Testing

The susceptibility testing of the clinical *Sporothrix* strain was determinate according to the Clinical and Laboratory Standards Institute (CLSI) through the M38-A2 broth microdilution protocol [24]. According to the M38-A2 protocol, a filamentous form of the *Sporothrix* strain was used. The inocula were prepared with fungal conidia in sterile saline solution after incubation of each strain on PDA for 7 days at 35 °C. The antifungal susceptibility tests were conducted according to the M38-A2 protocol of the CLSI, using RPMI 1640 medium buffered to pH 7.0 with 0.165 mol/L morpholinepropanesulfonic acid (Thermo Fisher Scientific^®^, Waltham, MA, USA), resulting in the final concentration of 0.4 × 10^4^ to 5 × 10^4^ CFU cells/mL, and incubation at 35 °C for 72 h.

Minimal inhibitory concentrations (MIC) were the lowest drug concentrations that produced either complete fungal growth inhibition for amphotericin B, itraconazole, and posaconazole. For ketoconazole, the MIC was the lowest concentration that produced a 50% reduction in growth, and for terbinafine it was the lowest concentration that produced at least an 80% reduction in growth. All the tests were performed in triplicate for the isolate. Tests were validated only if MIC values for these strains were within the range described in the M38-A2 reference document. MICs were determined by visual inspection after 72 h of incubation at 35 °C, as described (CLSI 2008). The reference strains *Aspergillus fumigatus* ATCC 204305 and *A. flavus* ATCC 204304 were included in each experiment for control.

### 2.4. Ethics Statement

This study was approved by the Research Ethics Committee of INI/Fiocruz (CAAE 28063114.2.0000.5262, approved on 24 April 2014). The patient was an adult and provided written informed consent. All data analyzed were anonymized.

## 3. Results

**Figure 1:**
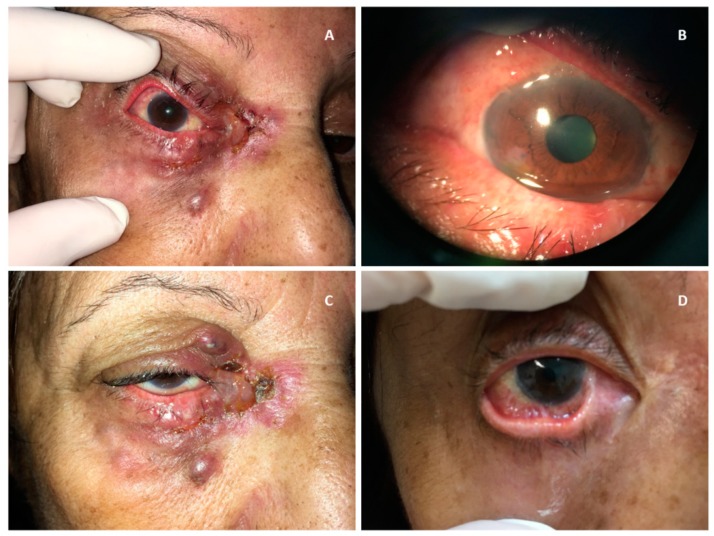
Lesions observed at the first clinical evaluation, (**A**) suppurative nodular lesions on eyelids and (**B**) subconjunctival infiltrative lesion occupying upper and lower fornices; (**C**) evolution of lesions in a short time interval, which increased in number and coalesced; (**D**) diffuse symblepharon after resolution of the acute phase, causing restriction of ocular abduction.

The patient presented with a subconjunctival infiltrative lesion forming an erythematous aspect throughout the ocular circumference of the right eye, occupying the upper and lower fornixes. She also presented with lower eyelid lesions, erythema, and thickening of the skin, ulceration in the inner corner of the eye, and ipsilateral pre-auricular lymphadenopathy. The left eye (LE) had no alterations (Figure 1A,B).

**Figure 2:**
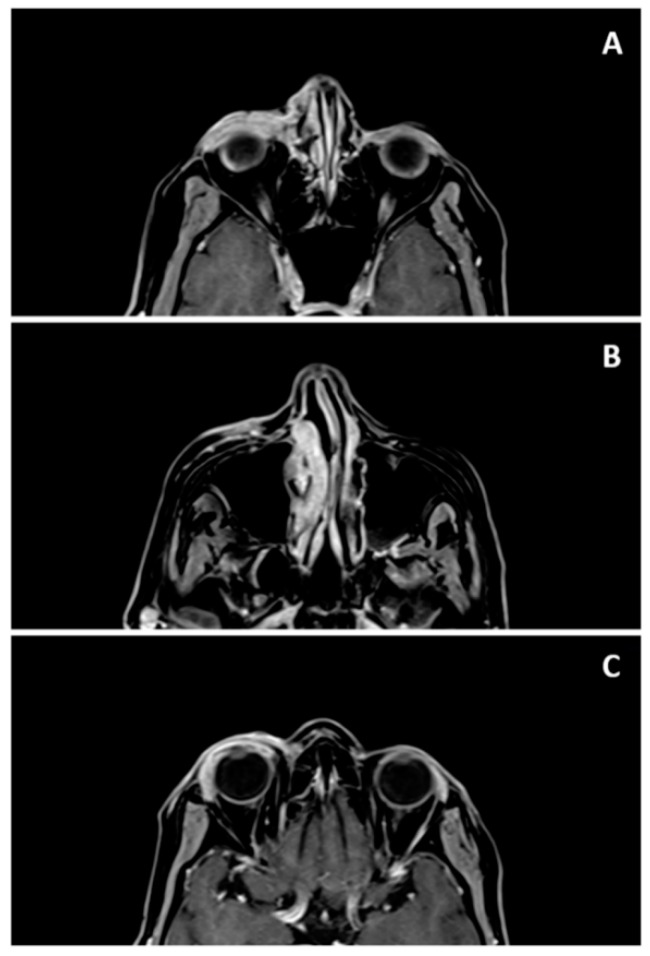
Nuclear Magnetic Resonance of orbits showing expansive lesion affecting: (**A**) conjunctiva and eyelids on the right side; (**B**) right inferior lacrimal system; (**C**) right lacrimal gland.

Nuclear Magnetic Resonance of the orbits revealed an expansive and infiltrative lesion involving the right eyelids and periorbital region extending to the ipsilateral lacrimal duct, with apparent involvement of the conjunctiva and lacrimal gland (Figure 2).

Bacterioscopy, direct examination with potassium hydroxide, and bacterial culture of the secretion collected from the conjunctival sac were all negative.

**Figure 3:**
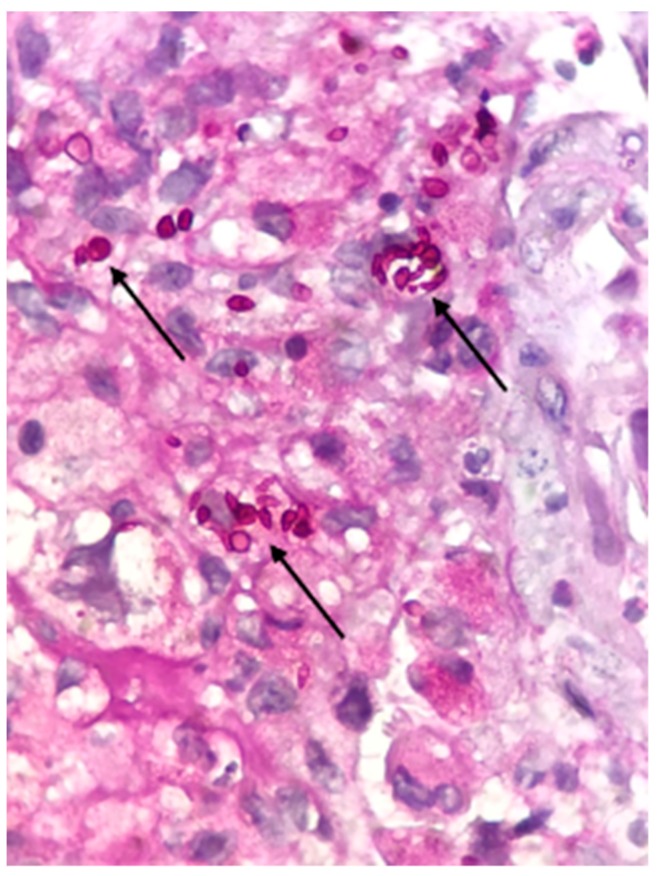
Histopathological stain with PAS of the bulbar conjunctiva (1000×)—arrows showing conspicuous round and oval yeasts within the granulomas.

A biopsy of bulbar conjunctiva showed a granulomatous inflammatory process associated with suppurative areas containing small round to oval bodies within giant cells and other mononucleated histiocytes. Grocott methenamine silver and Periodic Acid-Schiff (PAS) (both from Agilent, Santa Clara, CA, USA) highlighted these rather conspicuous yeasts (Figure 3).

Culture of the conjunctival secretion on Sabouraud Dextrose Agar showed fungal growth with raised moist colonies with a membranous aspect and a wrinkled or folded surface. Initially, the colonies were white to cream-colored, later turning brown to dark grey and black. Hyaline hyphae, septate and branched, with small conidiophores with claviform conidia implants in groups, were microscopically observed. It was then identified as *Sporothrix* sp. by traditional methodology through micro- and macromorphology, based on visual and light microscopy observations, respectively.

**Figure 4:**
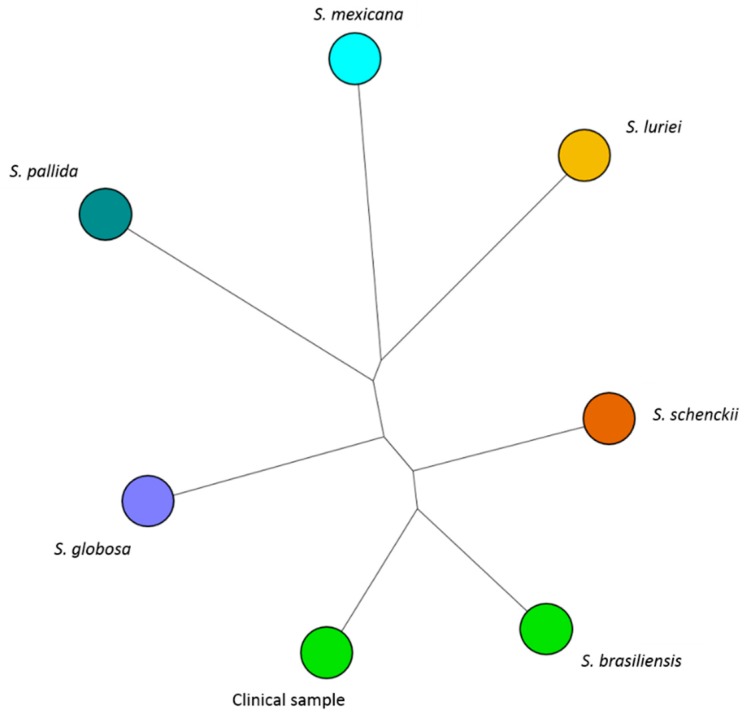
Neighbor-Joining tree based on Pearson correlation constructed with Matrix-Assisted Laser Desorption/Ionization Time-of-Flight Mass Spectrometry (MALDI-TOF MS) spectra of 7 *Sporothrix* isolates showing the 6 reference strains color-coded according to the *Sporothrix* species [*S. luriei* CBS 937.72 (Amber), *S. pallida* SPA8 (Teal), *S. mexicana* MUM 11.02 (Cyan), *S. schenckii* IPEC 27722 (Brown), *S. globosa* IPEC 27135 (Lavender) and *S. brasiliensis* CBS 120339/IPEC 16490 (Green)] and 1 clinical sample with color according to the *Sporothrix* species identified by MALDI-TOF MS.

Subsequently, the isolate was assessed by MALDI-TOF MS and acquired spectra were compared with reference spectra stored in an accurate in-house MALDI spectra reference database containing spectral data for the *Sporothrix* species complexes. The obtained spectra presented ion peaks compatible with the molecular mass of *Sporohtrix brasiliensis* (Figure 4).

While awaiting the results of the examinations, it was possible to observe the evolution of the skin lesions that became individualized, forming darkened nodules that suppurated (Figure 1C). After the diagnostic confirmation, in vitro susceptibility testing with amphotericin B, itraconazole, ketoconazole, posaconazole, and terbinafine were performed by the microdilution method [24] and revealed MICs of 1 µg/mL, 1 µg/mL, 0.25 µg/mL, 1 µg/mL and 0.125 µg/mL, respectively. According to the epidemiological cut-off values [25], this isolate was classified as a wild-type, thus justifying the initial antifungal treatment with amphotericin B 50 mg/day, subsequently replaced by itraconazole 200 mg/day.

The clinical condition evolved with noticeable improvement after the first week of treatment, with complete epithelialization of the ulcers after 1 month of medication. On the return visit, 3 months after the onset of the clinical condition, the patient presented with complete ptosis of the upper eyelid and symblepharon in the lower temporal region and upper and lower nasal regions, restricting ocular abduction (Figure 1D). The patient was treated with itraconazole 200 mg/day for a total period of 8 months. Treatment time was defined by the infectologist in charge of the case, who decided to keep the medication until the postoperative period of the symblepharon correction surgery.

The patient was then submitted to a symblepharon correction with a labial mucosa graft. After 8 months of symblepharoplasthy, the patient was evaluated for ptosis correction surgery that revealed severe ptosis with good levator muscle function in the affected eye. She was, therefore, submitted to reinsertion of the aponeurosis levator muscle of the upper eyelid.

**Figure 5:**
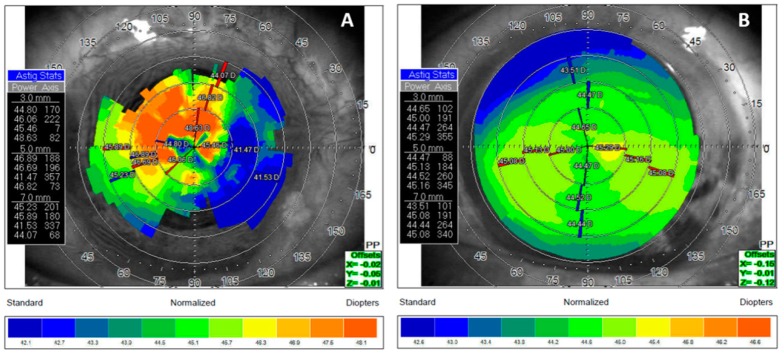
Comparative topography where (**A**) shows the right eye with irregular astigmatism caused by corneal scars and (**B**) shows the left eye with regular astigmatism.

Previously described surgeries have improved palpebral opening and ocular motility, but not all sequelae could be completely resolved with the surgery. A cicatricial epicantal fold was presented in the RE, with symblepharon recurrent in the nasal region, without diplopia in the primary position of gaze. Final corrected visual acuity was RE 20/80 (with rigid contact lenses) and LE 20/20. Low vision in the RE was probably due to residual astigmatism caused by scarring corneal irregularities and dry eye (Figure 5). Funduscopic examination revealed no abnormalities in both eyes.

## 4. Discussion

Sporotrichosis is a mycosis that predominantly affects the skin of the lower and upper limbs (hands, forearms, feet, and legs) and may also affect the face [26]. The eyes may be affected, with intraocular involvement, in the form of endophthalmitis, retinal granuloma, granulomatous necrotizing retinochoroiditis, and granulomatous uveitis, or the extraocular form, affecting tissues adjacent to the bulb and ocular adnexa [6,11,27,28].

Ramírez Soto found in a literature review of 16 publications related to endophthalmitis caused by sporotrichosis, 8 of which refer to exogenous endophthalmitis, an isolated form that occurs by direct ocular traumatic inoculation, and 10 cases of endogenous endophthalmitis, with hematogenous dissemination from other lesions [11]. In addition, the same author in a different study [6] observed 65 cases of patients with alterations only in the periocular appendages, from which the lymphocutaneous form was the most frequent, followed by the cutaneous-fixed form. However, other authors have described the fixed cutaneous form as the most prevalent, followed by lymphocutaneous [10,26].

The case herein described presented ocular involvement, with diffuse infiltration of the conjunctiva and corneal and extraocular alterations, with the involvement of the eyelids, lacrimal gland, and nasolacrimal duct, as evidenced by the imaging examination (Figure 2). The conjunctival and lacrimal sac involvement is even more rarely described [6] and the delay in the diagnosis of this patient may have contributed to the lesion extension. Other authors have also reported periods of 1–3 months until a definitive diagnosis is obtained [8,10]. Overall, the clinical presentation of the disease is variable [6] and can be misdiagnosed with other diseases such as hordeolum, lacrimal sac tumor [26], bacterial abscesses, and basal cell or sebaceous carcinoma [10] until a final diagnosis is obtained.

After the description of *Sporothrix schenckii* species complex by Marimon and collaborators [1], 150 cases of sporotrichosis with ocular involvement were published. Just 4.6% out of these cases had the isolation and fungal characterization at the species level (Table 1), being one case by *S. pallida* [29] and six other cases by *S. brasiliensis* [4,30,31,32]. However, in one of these studies [4], the fungus was not isolated from a clinical specimen recovered from the eyes, being disseminated disease presumed as a possible explanation to the diagnosis of ocular sporotrichosis associated to *S. brasiliensis*.

MALDI-TOF MS has been proven capable to identify fungal species belonging to the different fungal genus, as showed in previous studies available in the literature [16,17,18,20,21,22].

Oliveira and collaborators [16] successfully identified species of *Sporothrix* and have established an accurate in-house MALDI spectral database, which is comprehensive in terms of *Sporothrix* MALDI spectra number and geographical distribution of strain origins. Moreover, all reference spectra available in the mentioned in-house database are validated by molecular biological analysis.

In the present study, the use of MALDI-TOF MS associated with an in-house database enriched with reference *Sporothrix* complex spectra, in addition to the histopathology and morphology observations, were performed to obtain a reliable fungal identification at the species level. The isolated species, in this case, was *Sporothrix brasiliensis*.

As shown in a previous study [16], the MALDI-TOF MS technique is as good as the partial sequence of genes (calmodulin, beta-tubulin, and chitin synthase, for example) for the identification of *S. brasiliensis*. The report of sporotrichosis by *S. brasiliensis* corroborates epidemiological studies that describe the current high circulation of this species in Rio de Janeiro State, especially involving adult women who are out of the labor market [5,23,40].

The *Sporothrix schenckii* complex is found worldwide. Clinical cases have commonly been reported in geographical regions with high humidity and temperatures, which presents optimal conditions for fungal growth. However, cases of sporotrichosis have also been reported in temperate climate regions, such as European countries [5,41] and Chile [42]. Individual species of *Sporothrix* have presented different geographical distribution. *Sporothrix globosa* and *S. schenckii* have been reported from several locations in both Eastern and Western Hemispheres [43]. *Sporothrix mexicana* has been recovered from the environment in Australia, Mexico, and Portugal, where it has been reported to cause occasional human infections [41,43].

In contrast, *S. brasiliensis* seems to be restricted to Latin America, and as of 2017, it has been described only in Brazil, where it is responsible for an ongoing epidemic in cats, other animals, and humans [2,3,23,44]. In a review of human sporotrichosis reported in Rio de Janeiro State, da Silva and collaborators [40] reported two cases in the municipality of Paty do Alferes in the period from 1997 to 2007. Data obtained for the period from 2008 to 2017, from the Management of Vector-Transmitted Diseases and Zoonoses Sector of the Health Secretariat of Rio de Janeiro State, demonstrated two additional cases from Paty do Alferes, which were reported in the year 2012. It is worth noting that the sporotrichosis endemic is slowly spreading through the municipalities of Rio de Janeiro State and other Brazilian states [44,45].

An important factor in understanding the spread of this epidemic is the social vulnerability of these patients and the lack of adequate information for the population. In the case reported in this article, the patient herself reported that it was common practice in her neighborhood to bury cats that died with signs of injury, which leads to soil contamination and disease spread. In addition, there is a lack of a referral center for the care of these cases, especially in the inner cities, which leads to delayed diagnosis, with a greater chance of disease transmission and greater sequelae for affected patients. In this context, it is here emphasized the absence of fast methodologies for diagnosis of sporotrichosis, with the MALDI-TOF MS a good tool in the diagnosis of sporotrichosis, when a reliable spectral database is available.

## 5. Conclusions

The *S. brasiliensis* strain isolated from the subconjunctival infiltrative lesion of the patient was successfully identified using a MALDI-TOF MS associated with an in-house database enriched with reference *Sporothrix* complex spectra. Stablishing an in-house database is a good way to overcome limitation associated with the use of MALDI-TOF MS in fungal identification. The patient was adequately treated with amphotericin B subsequently replaced by itraconazole. Due to scars left by the suppurative process, the patient presented poor final visual acuity.

## Figures and Tables

**Table 1:** Cases of sporotrichosis with ocular involvement were published after the description of *Sporothrix schenckii* species complex.

Authors	Cases (*n*)	Identification Method	Species	References
Negroni et al., 2007	1	Culture and Histopathology	*Sporothrix* spp.	[33]
Inokuma, et al., 2010	1	Culture, Histopathology and Serology	*Sporothrix* spp.	[34]
Iyengar et al., 2010	1	Culture	*Sporothrix* spp.	[8]
Kashima et al., 2010	1	Histopathology and Serology	*Sporothrix* spp.	[12]
Silva-Vergara et al., 2012	1	Culture and Molecular	*S. brasiliensis*	[30]
Morrison et al., 2013	1	Culture and Molecular	*S. pallida*	[22]
Ferreira et al., 2014	1	Culture	*Sporothrix* spp.	[35]
Freitas et al., 2014	4	Culture	*Sporothrix* spp.	[36]
Zhang et al., 2014	1	Culture	*Sporothrix* spp.	[37]
de Macedo et al., 2015	1	Culture and Molecular	*S. brasiliensis*	[31]
Fan et al., 2016	10	Culture	*Sporothrix* spp.	[10]
Ramírez Soto, 2016	21	Culture	*Sporothrix* spp.	[6]
Zhang et al., 2016	72	Culture and Serology	*Sporothrix* spp.	[26]
Biancardi et al., 2017	3	Culture and Molecular	*S. brasiliensis*	[4]
Yamagata et al., 2017	3	Culture	*Sporothrix* spp.	[15]
Ling et al., 2018	1	Culture and Histopathology	*Sporothrix* spp.	[38]
Lacerda Filho et al., 2019	1	Culture and Molecular	*S. brasiliensis*	[32]
Arinelli et al., 2019	26	Culture	*Sporothrix* spp.	[39]
